# Family History of Colorectal Cancer: Clinicians' Preventive Recommendations and Patient Behavior

**Published:** 2011-12-15

**Authors:** Amy I. Zlot, Kerry Silvey, Nanette Newell, Ralph J. Coates, Richard Leman

**Affiliations:** Genetics Program, Public Health Division, Oregon Health Authority; Oregon Health Authority and Oregon Health and Science University, Portland, Oregon; Oregon Health Authority, Portland, Oregon; Centers for Disease Control and Prevention, Atlanta, Georgia; Oregon Health Authority, Portland, Oregon

## Abstract

Few population-based studies have addressed the role that family history of colorectal cancer (CRC) plays in clinician decision making or patient health choices. The objective of this study was to evaluate the effect of family history of CRC on clinician practice, patient CRC screening, and patient preventive behavior. We analyzed 2008 Oregon Behavioral Risk Factor Surveillance System data to examine associations between family history of CRC and 1) patient-reported clinician recommendations, 2) perceived risk of developing CRC, 3) adoption of preventive and screening behaviors, and 4) CRC risk factors among 1,795 respondents without CRC. A family history of CRC was positively associated with a higher likelihood of respondents reporting that their clinicians discussed colorectal cancer screening (OR, 4.2; 95% CI, 2.4-7.4) and of respondents having colorectal screening within the recommended time period (OR, 2.2; 95% CI, 1.3-3.9). A family history of CRC was also associated with respondents reporting lifestyle changes to prevent CRC (OR, 2.6; 95% CI, 1.7-4.0). A family history of CRC may prompt clinicians to recommend screening and preventive behavior changes and motivate patients to adopt such strategies.

## Introduction

Colorectal cancer (CRC) is a multifactorial disease, reflecting the interaction of hereditary and environmental factors. CRC was Oregon's fourth most common cancer and second leading cause of cancer death in 2008 (1). Although 50% to 75% of CRC can be prevented through detection and removal of precancerous polyps (personal communication, Lieberman DA, May 2008), CRC screening rates remain below 70% in Oregon (2). Family history of CRC is an independent risk factor for developing the disease.

**Box. Guidelines or Clinical Considerations Pertaining to Colorectal Cancer (CRC) Screening for People at Increased Familial Risk**

**Table:** 


**Organization**	**Recommendation/Clinical Consideration**	**Screening Test**
USPSTF	For high-risk people (eg, those who have a first-degree relative diagnosed with CRC at age <60 y), initiating screening at an earlier age (eg, age 50) is reasonable.	None specified.
ACS, the US Multi-Society Task Force on CRC, and the American College of Radiology	For people with CRC or adenomatous polyps diagnosed in a first-degree relative at age <60 or in 2 or more first-degree relatives at any age, begin screening at age 40 or 10 years younger than the youngest diagnosis in the family, whichever comes first.	Colonoscopy every 5 years. None specified.
	For people with CRC or adenomatous polyps diagnosed in a first-degree relative at age ≥60 or in 2 second-degree relatives with CRC, begin screening at age 40.	None specified.
NCCN	Recommends different screening frequencies and ages to begin screening based on the age and number of relatives diagnosed with CRC.	Colonoscopy is always preferred for people with a family history.
Abbreviations: USPSTF, US Preventive Services Task Force; ACS, Amercian Cancer Society; NCCN, National Comprehensive Cancer Network.		

Approximately 20% of CRC cases are associated with a family history, including 5% of CRC cases associated with single-gene cancer syndromes (3,4). People with a family history of CRC are 2.3 to 4.3 times more likely to develop CRC than those without it, depending on the number, relation, and age of onset of the relative(s) with cancer (5-7). People whose relatives have early-onset CRC (diagnosed before age 50) are at higher risk than those with relatives diagnosed later in life (8). Consequently, several organizations recommend early screening for people with a positive family history, depending on the age at which the relative was diagnosed (Box) (9-11).

Given the potential to reduce illness and death, early detection and prevention of CRC among people at high familial risk have important public health implications. These goals are achievable through clinician-mediated strategies (risk counseling, screening, or lifestyle modification recommendations) and patient-mediated strategies (risk-reducing lifestyle changes or use of screening). However, the frequency with which clinicians identify people with family history of CRC and provide them with preventive recommendations is poorly characterized (12). The objective of this study was to examine the relationship between family history of CRC and patient-reported clinician screening recommendations and patient preventive and screening behaviors among adults without CRC. We also examined associations of family history of CRC with other CRC risk factors and perceived risk of developing CRC.

## Methods

We used data from the 2008 Oregon Behavioral Risk Factor Surveillance System (BRFSS), a statewide, random-digit-dialed telephone survey of health conditions and risk behaviors of the noninstitutionalized Oregon population aged 18 years or older, to estimate family history of CRC, health care provider practice, CRC screening, and preventive behavior. Detailed information about the Oregon BRFSS is available elsewhere (13). We weighted the data by age and sex to better reflect the demographic characteristics of adults in Oregon. The Oregon Health Authority deemed projects that use BRFSS data, including this project, to be exempt from review.

### Survey measures

For this study, we classified respondents as having a family history if they reported at least 1 first-degree relative (parent, sibling, or child) with CRC and as having a family history of early-onset CRC if they reported at least 1 first-degree relative who was diagnosed with CRC before the age of 50. We classified respondents who reported no first-degree relatives with CRC or who reported they were adopted and did not know the medical history of their biological family members as having no family history.

Additionally, we collected information about respondent-reported clinician recommendations on CRC risk and screening, as well as discussions about prevention and behaviors that might affect CRC risk. We also collected information about whether or not patients acted on clinicians' recommendations.

We asked respondents, "Have you ever been told by a doctor, nurse, or other health care provider that you have colorectal cancer?" Respondents without CRC were asked the following questions:

"Thinking of your close blood relatives, do you have a parent, brother or sister, or child who has been diagnosed with colorectal cancer by a health care provider?" If they responded yes, they were asked to identify which of these relatives had been diagnosed with CRC and to report the number of relatives who had been diagnosed with CRC before the age of 50.

"Has a doctor, nurse, or other health care provider ever discussed the chances of you getting colorectal cancer?"

"Has a health care provider ever discussed testing for colorectal cancer with you?"

"Has a health care provider ever recommended changes in eating habits or physical activity to reduce your chances of getting diseases like colorectal cancer?"

"How likely do you think it is that you will get colorectal cancer in the future?"

"Have you made changes in your eating habits or physical activity to reduce your chances of getting diseases like colorectal cancer?"

Respondents without CRC and who were aged 50 or older were asked whether they had ever had a CRC screening test, including fecal occult blood test (FOBT)Respondents without CRC and who were aged 50 or older were asked whether they had ever had a CRC screening test, including fecal occult blood test (FOBT), sigmoidoscopy, or colonoscopy and the length of time since each test. We analyzed CRC screening prevalence by using 2 definitions: 1) ever having a colonoscopy, and 2) having CRC screening within the recommended time period based on the American Cancer Society and US Multi-Society Task Force on CRC guidelines for average-risk populations, which is either an FOBT within the past year, sigmoidoscopy within the past 5 years, or colonoscopy within the past 10 years (10).

### Potential covariates

We analyzed the following potential covariates, which may affect associations among family history, health care provider recommendations, and patient behavior. Covariates included self-reported information on age, sex, education level, annual household income, marital status, race/ethnicity, leisure-time physical activity within the past month, obesity, smoking status, alcohol use, insurance status, and having 1 person the respondent thought of as their personal doctor or health care provider. We defined obesity as having a body mass index of 30 kg/m^2^ or more, current smokers as people who reported smoking every day or some days and who reported having smoked 100 or more cigarettes during their lifetime, heavy alcohol use as consuming more than 2 alcoholic drinks per day on average for men and more than 1 alcoholic drink per day on average for women within the past month.

### Data analysis

We used Pearson χ^2^ tests and logistic regression to assess the association between respondents' family history status and reported health care provider practices, perceived risk for developing CRC, preventive and screening behaviors, and risk factors for CRC.

We included only covariates that were significantly associated with family history and the outcome variable in bivariate analyses in the multivariable logistic regression models. In the adjusted logistic regression models, we kept only covariates that changed the point estimate of the odds ratio (OR) by at least 10% (compared with the full model) in the final models. All analyses were performed using Stata version 11.0 (StataCorp LP, College Station, Texas). We reported sample sizes (number of survey respondents) as unweighted numbers and percentages as weighted estimates.

Of the 1,841 people who responded to the family history questions, we excluded 29 because of missing or unknown information about family history of CRC and 17 additional respondents who had CRC. Our final sample for this analysis included 1,795 respondents without CRC. Although we were able to present the overall prevalence of respondents with 2 or more relatives with CRC, we were unable to stratify this group by other variables because of the small number of respondents (n = 9).

## Results

The response rate for the Oregon 2008 BRFSS was 56%. Among respondents without CRC, 7.6% reported having a family history of CRC in first-degree relatives, and 1.1% were classified as having family history of early-onset CRC (Table 1). This translates into approximately 206,000 Oregonian adults without CRC with a positive family history and 30,000 Oregonian adults without CRC with family history of early-onset CRC (using a 2008 population estimate of 2.7 million Oregonian adults who had never been diagnosed with CRC). Of those with a family history, 4.6% (0.3% of the respondents without CRC) had 2 or more relatives with CRC.

Several demographic characteristics varied by family history status. A higher proportion of people aged 50 to 64 years had a family history of CRC compared with those aged 18 to 49 years (Table 1). Whereas 20.4% of college graduates reported having a family history, none of the respondents with fewer years of formal education reported having a family history of CRC. Other characteristics, including whether the respondent reported having a personal health care provider, did not differ by familial risk.

Respondents with a family history of CRC were more likely than those with no family history to report that their health care provider discussed the risk of developing CRC, discussed CRC screening, and recommended lifestyle changes to reduce the chance of developing CRC or other chronic conditions (Table 2). This result held true for the subset of respondents with family history of early-onset CRC.

Those with a family history of CRC were more than 8 times as likely to believe that they were at high risk for developing CRC in the future as people without a family history (Table 3). This result held true for those with a family history of early-onset CRC. Both those with any positive family history of CRC (OR, 2.6; CI, 1.7-4.0) and those with a family history of early-onset CRC (OR, 4.2; CI, 1.5-11.7) were more likely to report changing their eating and physical activity habits to decrease their risk of disease than were respondents with no family history. Having a family history of CRC was associated with current smoking but not with leisure-time physical activity, obesity, alcohol consumption, or health insurance coverage.

Among respondents aged 50 or older, those with a family history of CRC were more likely to report receiving screening for CRC within the recommended time frame for average-risk people than those without a family history (OR, 2.2; 95% CI, 1.3-3.9) (Table 3). Family history of early-onset CRC was a predictor of having ever had a colonoscopy (OR, 3.3; 95% CI, 1.0-10.7; *P* = .01). This result held true for the broader group of those with any family history of CRC (OR, 2.5; 95% CI, 1.5-4.2). Among respondents aged 50 or older who had a positive CRC family history, there was no significant difference in screening behavior between those with high perceived risk versus those with low perceived risk (data not shown).

## Discussion

Our study found an association between family history of CRC and CRC screening, consistent with previous studies (14-16). Awareness of family history could mitigate risk for developing CRC through multiple mechanisms. First, it could motivate a person at risk to adopt behaviors or seek screening that may help prevent the disease or diagnose it at an early stage when it is most curable (patient-mediated effects). Second, it could motivate clinicians to counsel patients with a CRC family history about their risk for the disease, strategies to decrease that risk, and appropriate screening (clinician-mediated effects). Third, it could motivate people to be more receptive to comments and recommendations of their health care providers.

### Patient-mediated effects, perceived risk, and behavior change

The strong association in this study between family history of CRC and perceived risk of developing the disease is consistent with some prior studies involving other diseases (14-16), although the literature is inconsistent. However, as shown in this and other studies, there is no guarantee that high perceived risk will translate into risk-mitigating behavior. In a study of women with a family history of breast cancer, Drossaert et al noted that although these women had higher perceived risk, there were no differences in early-detection behavior between women with or without family histories of the disease (14). Some studies have suggested that heightened perceived risk leads to fatalism and actually impedes adaptive behavior to decrease risk (17,18). Other research has demonstrated that having a close family member with a chronic disease is associated not only with increased perception of risk but also with preventive and health-promoting behaviors (19-21). Another study of people who had first-degree relatives with CRC found that family history, perceived risk, and the belief that CRC can be prevented all increased the odds of CRC screening (22). Among respondents aged 50 or older in our study, whereas family history was associated with higher rates of screening, higher perceived risk was not. This could mean that simply being aware of one's family history is a motivator for screening, and other preventive behaviors are independent of perceived risk. It is possible that other factors, such as interactions with health care providers, exert a stonger influence on screening behaviors (22).

None of the respondents with less than a college education reported having a family history of CRC. It is unlikely that these people were entirely without family members diagnosed with CRC. This finding may reflect a difference in health literacy and awareness of the potential influence of family history on one's personal health between college graduates and those with fewer years of formal education (23,24). Clinician assessment of health literacy in this area, followed by counseling about the potential value of family history information for patients who report no knowledge of their family history, could help address this disparity.

### Clinician-mediated effects

Although prior studies have demonstrated that clinicians can be effective catalysts for behavior change in their patients (15,16), our study is the first of its kind to show a positive association between family history of CRC and reported discussion by clinicians about CRC screening and lifestyle changes intended to reduce disease risk. The literature is less clear about the extent to which clinicians collect family history information about screenable malignancies, such as CRC, and whether they then use this information to drive discussions with their family history–positive patients about familial risk and tailor subsequent screening recommendations.

Those in our study with a family history of CRC were significantly more likely to report that their health care provider had discussed their risk of developing CRC with them and had recommended screening. This finding suggests that clinicians, once aware of relevant family history, appear to incorporate it into risk stratification, patient education, and screening decisions.

Still, the low absolute percentages of respondents with a positive family history reporting that a health care provider engaged in discussions about risk of CRC or that they received recommendations on strategies to prevent CRC suggest many missed opportunities to educate people at increased risk of CRC and steps that could be taken to detect and prevent the condition.

Incorporating family history into clinical practice is made more challenging by the absence of widely accepted standards for its collection and use (19). Although the use of family history tools on risk assessment and health outcomes has yet to be thoroughly researched, the authors of 1 review concluded that family history collection tools could add better family medical information compared with current primary care practice (25).

This study has several limitations. First, causal inferences cannot be drawn from our cross-sectional data. Second, because survey data were self-reported, they are subject to recall bias. However, research has shown that most measures on the BRFSS are reliable and valid (26). Also, some studies have shown that people can accurately report their family history of CRC (27), whereas others indicate that family history of CRC is underreported (28). This is especially pertinent to our study because none of the respondents with less than a college education reported having a family history. Third, the response rate (56%), though well above the median for BRFSS response rates for all of the states, may have introduced nonresponse biases that affected the inferences drawn from this study. Fourth, BRFSS asked about screening for CRC only among respondents aged 50 or older. Consequently, we were unable to determine whether younger people with a positive family history were being screened according to guidelines for people at high familial risk. Fifth, having a family history of CRC could affect how people perceive and interact with their clinicians. For example, people with a family history of CRC may be more likely to recall that their clinician made recommendations and discussed risk for developing CRC and screening options, whereas people with no family history may be more likely to forget these interactions. Sixth, although we did capture information on first-degree relatives with CRC and the age of onset of the disease, we did not ask about second- or third-degree relatives. Lastly, clinician behavior was reported by the respondents in the survey and was not actually observed or reported by the clinicians themselves.

### Implications for public health policy and future research

The finding that having a family history of CRC is associated with higher rates of screening for this condition could be due to personal motivation, mediated by health care provider counseling and screening recommendations, or a mixture of both. Although people with a family history of CRC were more likely to report clinician risk counseling and recommendations to undertake screening, the absolute percentages receiving such counseling and recommendations were low. Development and dissemination of evidence-based, systematic clinician training could increase clinicians' ability to collect CRC-related family history information and improve their awareness of how to use that information most effectively in counseling and screening. Clinician training could also provide necessary skills to assess patient health literacy in this area and, subsequently, help those with limited knowledge of their family history gain a better understanding of their risk of disease.

Universal, timely screening for CRC among people at increased risk due to family history would prevent disease and save lives. To accomplish this goal, clinicians require efficient decision support tools and consistent evidence-based guidelines related to family history. Future translational studies are needed that facilitate use of family history information to motivate clinicians and their patients to pursue appropriate CRC screening.

## Acknowledgments

This project was supported by the Centers for Disease Control and Prevention (CDC) Cooperative agreement no. CDC-RFAGD08- 801 (grant no. 1U38GD000061).

## Figures and Tables

**Table 1:** Family History of CRC, by Sociodemographic Characteristics, 2008 Oregon Behavioral Risk Factor Surveillance System

**Characteristic**	**n** a	No Family History^b^% (95% CI)	Any Family History^c^% (95% CI)	Early-Onset Family History^d^% (95% CI)
**Total**	1,795	92.4 (90.9-93.7)	7.6 (6.3-9.1)	1.1 (0.7-1.8)
**Age, y**				
18-49	626	94.5 (92.2-96.2)	5.5 (3.8-7.8)	0.7 (0.3-1.8)
50-64	625	89.2 (86.4-91.5)	10.8 (8.5-13.6)	1.5 (0.7-2.8)
≥65	538	90.4 (87.2-92.8)	9.6 (7.2-12.8)	2.0 (1.0-4.2)
**Sex**				
Male	696	93.3 (91.0-95.1)	6.7 (4.9-9.0)	0.8 (0.3-1.7)
Female	1,099	91.6 (89.5-93.3)	8.4 (6.7-10.5)	1.5 (0.9-2.6)
**Education**				
High school or less	587	587 (100)	0	0
Some college	535	535 (100)	0	0
College graduate	663	79.6 (75.8-83.0)	20.4 (17.0-24.2)	3.3 (2.1-5.2)
**Marital status**				
Not married	670	94.1 (91.4-96.1)	5.9 (3.9-8.6)	1.3 (0.5-3.2)
Married	1,119	91.7 (89.8-93.3)	8.3 (6.7-10.2)	1.0 (0.6-1.7)
**Rurality**				
Urban	1,348	92.6 (90.9-94.0)	7.4 (5.9-9.1)	1.2 (0.7-2.0)
Rural	447	92.0 (88.5-94.5)	8.1 (5.6-11.5)	1.0 (0.5-2.3)
**Annual household income, $**				
<25,000	374	92.9 (89.4-95.4)	7.1 (4.6-10.6)	1.8 (0.8-4.7)
25,000-49,999	508	91.5 (88.3-93.8)	8.5 (6.2-11.7)	1.3 (0.7-2.7)
≥50,000	708	92.2 (89.7-94.1)	7.8 (5.9-10.3)	1.0 (0.5-2.0)
**Personal doctor/health care provider^e^**				
No	255	94.6 (90.7-96.9)	5.4 (3.0-9.3)	1.2 (0.3-4.0)
Yes	1,535	91.8 (90.1-93.2)	8.2 (6.8-9.9)	1.2 (0.8-2.0)

Abbreviations: CRC, colorectal cancer; CI, confidence interval.

a Numbers for some variables do not total 1,795 because of missing data.

b No first-degree relatives diagnosed with CRC or adopted with unknown family history status of blood relatives.

c One or more first-degree relatives diagnosed with CRC.

d One or more first-degree relatives aged <50 y diagnosed with CRC.

e Reported having 1 person whom the respondent thought of as his or her personal doctor or health care provider.

**Table 2:** Prevalence and Odds Ratios for Clinician Practices and Recommendations to Prevent CRC, by Family History of CRC, 2008 Oregon Behavioral Risk Factor Surveillance System

**Practice/Recommendation**	No Family History^a^% (95% CI)	Any Family History^b^% (95% CI)	Early-Onset Family History^c^% (95% CI)	Any Family History^b^OR (95% CI)(vs No Family History)	Early-Onset Family History^c^OR (95% CI)(vs No Early-Onset Family History)
**Discussion of CRC risk**					
No	88.5 (86.8-90.0)	51.7 (42.5-60.9)	50.7 (28.8-72.3)	1 [Reference]	1 [Reference]
Yes	11.5 (10.0-13.2)	48.3 (39.1-57.5)	49.3 (27.7-71.2)	8.9 (5.5-14.3)^d^	5.3 (2.0-13.9)^d^
**Discussion of CRC screening**					
No	70.5 (68.0-72.9)	36.8 (26.6-46.1)	26.4 (9.3-55.6)	1 [Reference]	1 [Reference]
Yes	29.5 (27.1-32.0)	63.2 (52.7-72.5)	73.6 (44.4-90.7)	4.2 (2.4-7.4)^e^	6.7 (1.6-27.8)^f^
**Lifestyle change recommendations^g^**					
No	89.6 (84.8-88.7)	69.2 (60.5-76.7)	52.7 (30.7-73.7)	1 [Reference]	1 [Reference]
Yes	13.1 (11.3-15.2)	30.8 (23.3-39.5)	47.3 (26.3-69.3)	2.6 (1.7-4.1)^f^	4.2 (1.5-11.7)^f^

Abbreviations: CRC, colorectal cancer; CI, confidence interval; OR, odds ratio.

a No first-degree relatives diagnosed with CRC or adopted with unknown family history status of blood relatives.

b One or more first-degree relatives diagnosed with CRC.

c One or more first-degree relatives aged <50 y diagnosed with CRC.

d Adjusted for education.

e Adjusted for education and age.

f Adjusted for education, age, and smoking status.

g Health care provider ever recommended changes in eating habits or physical activity to reduce the chances of getting diseases such as CRC.

**Table 3:** Prevalence and Odds Ratios for Perceived Risk, Risk Factors, and Screening Behaviors for CRC, by Family History of CRC, 2008 Oregon Behavioral Risk Factor Surveillance System

**Characteristic/Behavior**	No Family History^a^% (95% CI)	Any Family History^b^ % (95% CI)	Early-Onset Family History^c^% (95% CI)	Any Family History^b^OR (95% CI)(vs No Family History)	Early-Onset Family History^c^OR (95% CI)(vs No Early-Onset Family History)
**Perceived risk of CRC**					
Low^d^	92.5 (90.6-94.1)	54.2 (44.3-63.8)	44.7 (23.4-68.1)	1 [Reference]	1 [Reference]
High^e^	7.5 (5.9-9.4)	45.8 (36.2-55.7)	55.3 (31.9-76.6)	8.5 (4.6-15.7)^f^	8.7 (3.1-24.1)^f^
**Reported lifestyle changes^g^**					
No	67.7 (64.9-70.5)	43.4 (34.2-53.0)	29.0 (12.9-53.0)	1 [Reference]	1 [Reference]
Yes	32.3 (29.5-35.1)	56.6 (47.0-5.8)	71.0 (47.0-87.1)	2.6 (1.7-4.0)^h^	4.2 (1.5-11.7)^f^
**Any leisure-time physical activity within the past month**					
No	20 (17.4-22.8)	17.8 (11.9-25.7)	28.8 (12.8-52.8)	1 [Reference]	1 [Reference]
Yes	80 (77.2-82.6)	82.2 (74.3-88.1)	71.2 (47.2-87.2)	0.9 (0.5-1.4)	1.5 (0.6-4.6)
**Obesity (BMI >30 kg/m^2^)**					
No	73.1 (70.0-76.0)	70.2 (60.8-78.2)	58.5 (34.0-79.4)	1 [Reference]	1 [Reference]
Yes	26.9 (24.0-30.0)	29.8 (21.8-39.2)	41.5 (20.6-66.0)	1.2 (0.7-1.8)	1.9 (0.7-5.3)
**Current smoker**					
No	86.4 (84.0-88.4)	75.1 (64.4-83.3)	77.1 (47.0-92.7)	1 [Reference]	1 [Reference]
Yes	13.6 (11.6-16.0)	24.9 (16.7-35.6)	22.9 (7.3-53.0)	2.3 (1.4-4.0)^i^	1.9 (0.5-7.2)
**Heavy alcohol use^j^**					
No	95.5 (94.1-96.5)	92.2 (84.7-96.2)	100	1 [Reference]	1 [Reference]
Yes	**73 (3.5-5.9)**	**11 (3.8-15.3)**	0	1.5 (0.7-4.0)^k^	NA
**Health insurance coverage**					
No	16.6 (14.0-19.6)	10.2 (5.0-19.7)	17.0 (3.9-50.5)	1 [Reference]	1 [Reference]
Yes	83.4 (80.4-86.0)	89.8 (80.3-95.0)	83.0 (49.5-96.1)	0.5 (0.2-1.0)^k^	1.2 (0.3-5.3)^h^
**Colonoscopy ever^l^**					
No	46.3 (42.9-49.7)	23.3 (16.3-32.2)	16.7 (6.0-38.6)	1 [Reference]	1 [Reference]
Yes	53.7 (50.3-57.1)	76.7 (67.8-83.7)	83.3 (61.4-94.0)	2.5 (1.5-4.2)	3.3 (1.0-10.7)
**CRC screening^l^^,^ m**					
No	36.8 (33.5-40.3)	16.8 (11.0-24.9)	12.3 (3.8-33.3)	1 [Reference]	1 [Reference]
Yes	63.2 (59.7-66.5)	83.3 (75.1-89.0)	87.7 (66.7-96.2)	2.2 (1.3-3.9)^n^	2.4 (0.7-8.8)^n^

Abbreviations: CRC, colorectal cancer; CI, confidence interval; OR, odds ratio; BMI, body mass index; NA, not applicable.

a No first-degree relatives diagnosed with colorectal cancer or adopted with unknown family history status of blood relatives.

b One or more first-degree relatives diagnosed with colorectal cancer.

c One or more first-degree relatives aged <50 y diagnosed with colorectal cancer.

d Reported very or somewhat unlikely or neither likely nor unlikely to develop CRC in the future.

e Reported very or somewhat likely to develop CRC in the future.

f Adjusted for education.

g Reported making changes in eating habits or physical activity to reduce the chances of getting diseases such as colorectal cancer.

h Adjusted for age and smoking status.

i Adjusted for age.

j Defined as >2 drinks per day on average for men and >1 alcoholic drink per day on average for women within the past month.

k Adjusted for smoking status.

l  Among respondents aged ≥50.

m Fecal occult blood testing within the past year, sigmoidoscopy within the past 5 y, or colonoscopy within the past 10 y.

n Adjusted for education and smoking status.
